# The Role of VEGF in Intervention-Mediated Injuries: Neointimal Hyperplasia and In-Stent Restenosis

**DOI:** 10.3390/jcm14176184

**Published:** 2025-09-01

**Authors:** Amun G. Hofmann

**Affiliations:** FIFOS—Forum for Integrative Research & Systems Biology, 1170 Vienna, Austria; amungeorg.hofmann@gesundheitsverbund.at

**Keywords:** VEGF, intima hyperplasia, angioplasty, neointimal hyperplasia

## Abstract

**Background:** The role of vascular endothelial growth factor (VEGF) in intimal hyperplasia has been investigated and discussed numerous times in the literature, producing contrary results and controversial outcomes. In particular, research concerned with the effects of VEGF after catheter-mediated injuries regarding the development of neointimal hyperplasia resulted in diverging conclusions. **Methods:** A systematic review based on PRISMA principles using MEDLINE was conducted. In summary, 66 publications met the qualifying criteria to be included in this review. **Results:** VEGF can both cause and attenuate neointimal hyperplasia depending on its site of application and production. Endogenous VEGF produced in the media and adventitia promotes intimal hyperplasia after vascular injury, while exogenous VEGF delivered through drug eluting-stents or by gene therapy can ameliorate re-endothelialization and thereby inhibit intima hyperplasia. **Conclusions:** The understanding of post-injury released cytokines such as VEGF holds great promise for currently used therapeutic applications and potential for applications to be investigated in the future.

## 1. Introduction

Vascular endothelial growth factor (VEGF), often specifically also referred to as VEGF-A, is the main regulator of angiogenesis and permeability in blood vessels (and therefore historically it is also called vascular permeability factor). It is part of the platelet-derived growth factors and encompasses VEGF-A, VEGF-B, VEGF-C, VEGF-D and placental growth factor [1,2,3,4,5]. Additionally, viral and snake venom homologs have been found (VEGF-E and VEGF-F), but their nomenclature is based on molecular resemblance and physiological activity and they are often separated from the VEGF family [6,7]. Up to nine different VEGF-A isoforms that differ at the amino acid sequence have been described in humans [8]. VEGF gene expression is up-regulated by a plethora of cytokines and growth factors such as hypoxia-inducible factor-1α, which is repeatedly regarded as the most effective up-regulator [9,10]. Other factors include platelet-derived growth factor-BB, fibroblast growth factor, epidermal growth factor, tumor necrosis factor, transforming growth factor-β, interleukin-1, and insulin-like growth factor-1 [5,11].

**Figure 1:**
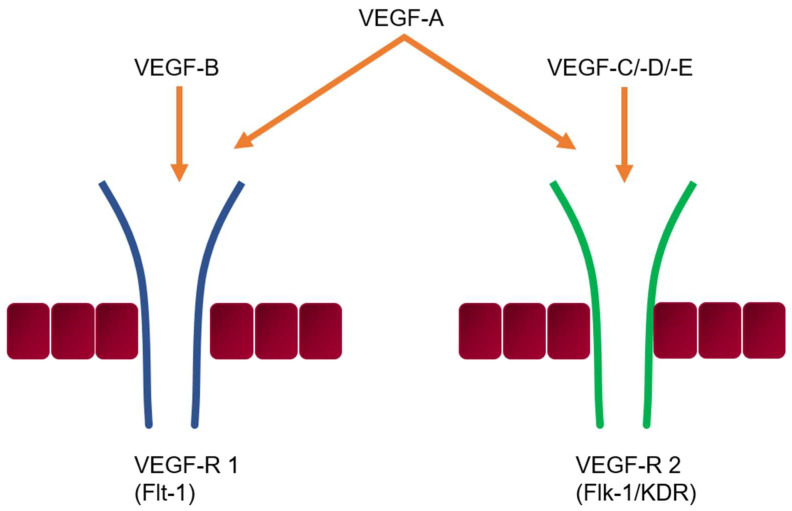
Members of the vascular endothelial growth factor (VEGF) family and their receptor binding patterns.

The receptor-binding pattern of VEGF is distinguishable from other members of the growth factor family, showing an affinity for VEGF receptor-1 (VEGFR-1; also called Flt-1) and VEGF receptor-2 (VEGFR-2; also called KDR) (Figure 1). Physiologically, VEGF is the key contributor to angiogenesis through a direct mitogenic effect on endothelial cells while also inducing anti-apoptotic signals, without which endothelial cells undergo apoptosis in immature vasculature [12,13]. Furthermore, VEGF is additionally involved in increasing vascular permeability, especially in microvessels [8].

Besides its physiological activity, VEGF is also the key mediator of angiogenesis in malignant tumors, whereby it is up-regulated by oncogene expression and hypoxia to result in distinct tumor vasculature that allows for exponential growth [14]. Therefore, VEGF became an attractive target for antineoplastic therapies such as the humanized monoclonal antibody bevacizumab [11]. However, their efficacy and optimal inclusion in established treatment regimens is still under investigation [15].

The inner layer of blood vessels, the tunica intima, consists of endothelial cells, and in the case of elastic arteries is surrounded by tunica media, a layer of elastin-enriched collagen and smooth muscle cells (SMCs), also referred to as myointimal cells. A thickening of the intima is attributed to the migration and proliferation of SMCs of the tunica media [16]. The rather general term of intimal hyperplasia is specified as neointimal hyperplasia in arteries secondary to injury, often associated with angioplasty [17].

Endovascular interventions frequently cause injuries during balloon dilation or stent implantation that subsequently induce SMC proliferation and migration, leading to a restenosis in treated segments [18]. Restenosis is a multifactorial process characterized by an interplay of inflammatory, proliferative and coagulative factors, which limits the long-term effectiveness of endovascular interventions [19]. While SMCs are the focal cell type, it should be stated that multiple interactions within the local environment shape the course of neointimal hyperplasia, ranging from adventitial fibroblasts [20] to extracellular vesicles [21]. Understanding neointimal hyperplasia as the primary pathomechanism behind restenosis is a critical task in improving patient care in cardiovascular disease. In coronary interventions, historic rates ranged from 32 to 55% for pure angioplasties, whereas bare metal stents and subsequently drug-eluting stents and drug coated balloons decreased its occurrence to less than 10% [22]. Results and developments have been similar in peripheral arterial-occlusive disease, from restenosis rates of 40–60% in the balloon-only era to less than 10% in drug-releasing materials [23].

Treatment and prophylaxis of intimal hyperplasia historically focused on preventing NO-deficiency and anti-inflammatory therapy. Newer approaches include graft seeding with tropoelastin or interference in the RhoA/ROCK signaling cascade, which causes mesenchymal stem cells to differentiate into myofibroblasts that contribute to intimal hyperplasia [24,25]. With expanding sets of delivery platforms and the introduction of further immunomodulatory agents, it might be feasible to further decrease restenosis rates and improve clinical outcomes after endovascular interventions [23].

As a key contributor to angiogenesis and more specifically because of its effects on endothelial and vascular SMCs (VSMC), VEGF-A has been under investigation for almost two decades as a potential target to understand and combat post-interventional neointimal hyperplasia. However, diverging reports on its effects complicated the establishment of a final conclusive answer. The present review aims to provide a comprehensive overview regarding the role of VEGF-A (hereafter also simply referred to as VEGF) on neointimal hyperplasia and restenosis as a consequence of intervention-mediated arterial injuries.

## 2. Methods

The present project is a systematic review of the literature regarding the role and function of VEGF in intervention-mediated arterial injuries.

### Literature Research

**Figure 2:**
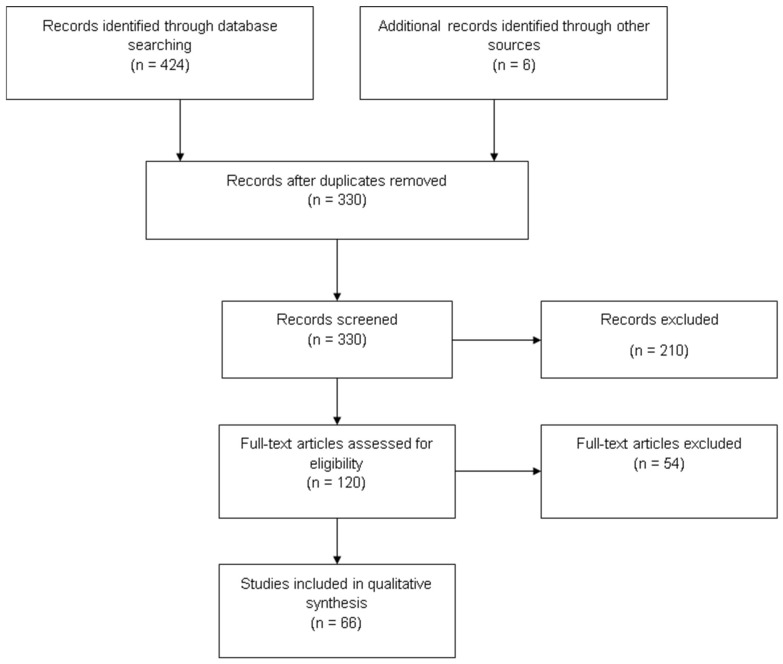
Flowchart of the literature research regarding VEGF and neointimal hyperplasia based on PRISMA.

A PubMed (MEDLINE) search was conducted in January 2025 using the query terms “VEGF neointimal hyperplasia”, “VEGF intimal hyperplasia”, “VEGF intima hyperplasia” and “VEGF intima thickening”. The publications were analyzed based on PRISMA (see Figure 2 for flow chart), resulting in 66 publications that were included in the final qualitative analysis. Original research papers were deemed relevant only under the condition that they produced either a direct relationship between VEGF and intimal thickening or could establish/explain a cascade that related intimal hyperplasia to VEGF levels through comprehensible reasoning and scientific proof in the context of vascular injury and/or catheter interventions. The review was not registered. Supplementary Table S1 illustrates a brief overview of included publications.

Furthermore, a MEDLINE search using the query “vegf vascular smooth muscle cells” was conducted by means of a mini review, resulting in an additional 11 publications, to depict the topic appropriately and assemble a fundamental summary.

## 3. Results

### 3.1. VEGF and Vascular Smooth Muscles Cells

The effects of VEGF-A on VSMCs are inextricably linked with its role in intimal hyperplasia. The historic process of scientific findings primarily concluded that VEGF-A was unable to induce proliferation in vascular SMCs, but revealed a function on migration. The mitogenic activity of VEGF was supposed to be endothelial cell-specific, even though synergistic effects of VEGF in FGF-2-induced VSMC proliferation were observed [26,27,28]. VEGF treatment was shown to enhance the production of matrix metalloproteinase (MMP)-1, -3 and -9, which may also contribute to SMC migration [27]. It was further revealed that at injured sites, SMCs themselves on the one hand are the main cellular source of VEGF, but on the other hand are more responsive to its effects through the induction of functional VEGFR-1 [29,30]. The autocrine production of VEGF is also at least partially responsible for the VSMC migration triggered by the monocyte chemoattractant protein 1 (MCP-1), a member of the CC chemokine family [31]. Furthermore, the migration of VSMCs was more and more associated with the effects of VEGF. In atherosclerotic plaques, T-cell-released interleukin-3 triggered not only SMC DNA synthesis, but also VEGF gene transcription and SMC migration [32]. Thrombin-induced migration also correlated with a higher VEGF expression [33]. While the relationship between VEGF and VSMC migration was established by consensus, by the mid-2000s, previously abandoned discussion of its proliferative effects had resumed. Exogenous VEGF was shown to have a vascular protective function by directly affecting VSMC proliferation via inhibition of the MAP-kinase pathway [34]. On the contrary, Parenti et al. attributed the mitogenic effects of MCP-1 to endogenous VEGF-A, while Osada-Oka et al. reported that hypoxia-induced proliferation of coronary artery SMCs is mediated through an autocrine mechanism by the expression of VEGF and VEGFR-1 [31,35]. However, in a cyclic strain model that caused an antimitogenic response in VSMCs, the increased secretion of NO and VEGF corresponded with a decrease in proliferation, while exogenous VEGF in the absence of strain did not inhibit proliferation [36]. In summary, the effects of VEGF on VSMC migration are well researched, while its proliferative function might depend on further factors from the local environment.

### 3.2. Endogenous VEGF Activity and Arterial Injuries

In periadventitial injury, VEGF was believed to promote neointimal formation by acting as a proinflammatory cytokine, while its effects could be inhibited by transfection with the murine Flt-1 gene [37]. In intraluminal injuries, such as, for example, those caused in endovascular interventions, increased expression and activity of VEGF was shown to be essential in the development of restenosis by recruiting monocytes, which could be attenuated by soluble Flt-1 [38,39]. Additionally, VEGF was found to promote neointimal hyperplasia through Flk-1 by regulating both MCP-1 expression in VSMCs and macrophage-mediated inflammation [40]. Examination of porcine coronary artery stents suggested that VEGF involvement in restenosis could be attributed both to angiogenic properties and synergistic effects with PDGF [41]. Physiological up-regulation of endogenous VEGF expression was also found to be incapable of inhibiting intimal thickening [42]. Additionally, ATF-4 expression is induced in smooth muscle cells in response to injury and stimulated through FGF-2. ATF-4 itself up-regulates VEGF transcription in vascular smooth muscle cells and mediates intimal thickening [43]. When VEGF plasma levels before and after PCI were correlated with in-stent restenosis (ISR) in 85 drug-eluting stent treated patients, it was observed that an increase in VEGF after PCI is significantly correlated with subsequent lumen loss during an eight-month follow-up period [44]. However, regarding late (1–4 years) and very late (≥5 years) ISR, serum VEGF levels showed an inverse relationship with impaired patency [45]. The beneficial effects attributed to immunosuppressive sirolimus (also known as rapamycin) eluting stents on ISR are widely accepted, and their usage results in reduced VEGF expression in SMCs [46,47,48] and peripheral monocytes [49]. Combined with the observations that granulocyte colony-stimulating coated stents induced neointimal hyperplasia [47], proinflammatory M1 macrophages correlate with VEGF expression and neointima formation [50], and the finding of increased interleukin-6 (IL-6) and VEGF levels in restenosis after PCI [51], this suggests that post-interventional lumen loss is also a VEGF-mediated inflammatory response. Adventitial fibroblast-derived VEGF causes an increase in vasa vasorum count, which in turn serves as a delivery platform for macrophages to injured sites [52]. Accordingly, high-nitrogen low-nickel coronary stents reduced in-stent restenosis by inhibiting inflammation as evidenced by decreased IL-6 and VEGF expression in vivo [53]. Perivascular down-regulation of thrombospondin-2 also led to a relevant decrease of neointimal formation that was accompanied by a decrease in VEGFR-2, highlighting again the effects of adventitial VEGF on intimal hyperplasia [54].

The association of endogenous VEGF and intima hyperplasia was further indirectly shown by reduced neointima formation in bevacizumab-eluting stents, a monoclonal antibody specific for VEGF, and after local thalidomide application, which acts as a VEGF inhibitor [55,56]. While the effects of estrogen as an inhibitor of neointimal thickening are repeatedly confirmed, controversial data regarding the associated occurrence of VEGF exist, with both increased [57] and decreased [58,59] levels being described. More recent studies regarding an association between genetic polymorphisms and in-stent restenosis in human coronary artery stents show that specific VEGF-A genotypes (among other growth factors) are associated with in-stent restenosis and late lumen loss more frequently than others [60,61,62]. Rather recently, investigations regarding the beneficial effects of miR-126 on neointimal formation were shown to be mediated by activating the VEGF signaling pathway [63,64]. Endogenous VEGF has therefore been repeatedly associated with neointimal hyperplasia and ISR as part of an inflammatory response to injury.

### 3.3. VEGF-Based Gene Therapy

On one hand, Khurana and colleagues observed that after local arterial wall injury, the application of VEGF-A to the adventitia induced a marked increase in neointimal thickening, and reached the conclusion that adventitial angiogenesis stimulates intimal thickening even though it does not initiate it [65]. On the other hand, Pels and colleagues showed that catheter-mediated (peri)adventitial VEGF165 gene transfer induced adventitial neovascularization, but not an increase of vascular thickening in a porcine model of coronary artery injury [66]. A possible explanation is the resulting inhibition of adventitial microvessel regression [67]. Asahara et al. investigated an inhibiting effect of site-specific arterial gene transfer of phVEGF165, which additionally accelerated re-endothelialization [68]. Accordingly, adventitial transfection with adenoviral encoding soluble VEGF-R 1 and 2 leads to reduced ISR rates, which was hypothesized to counter adventitial neovascularization mediated by endogenous VEGF [69].

The attenuation of neointimal hyperplasia and acceleration of re-endothelialization through the endovascular arterial gene transfer of both viral and plasmid DNA encoding VEGF was shown in multiple studies leveraging different animal models [70,71,72,73,74,75,76,77]. While a contrary report does exist [78], it should be stated that its authors suggest that the amount of VEGF produced by the plasmid vector was very low. These findings indicate that (peri)adventitial VEGF acts as a hyperplastic stimulus while the endoluminal VEGF increases re-endothelialization rates which limits hyperplasia and ISR.

Concordant with these findings are results generated by local transfection for the VEGF receptor KDR/flk-1, which also attenuated neointimal formation and luminal narrowing [79]. While early VEGF transfection models were primarily based on viral vectors, more recent studies validated the beneficial effects of VEGF gene therapy on intimal thickening through nanoparticle delivery, thereby eliminating adverse effects associated with viral vectors [80,81,82]. Hutter and colleagues showed that VEGF overexpression could accelerate endothelial repair and inhibited neointima formation after arterial injury, while the sequestration of exogenous and endogenous VEGF by VEGF-trap not only delayed re-endothelialization but also increased neointima size [83]. These findings were confirmed through the delivery of a plasmid vector with the VEGF gene specifically to the injury site through the endothelial cell-targeting CDG2-cRGD-5 polyplex [42]. Nevertheless, most evidence stems from in vitro and animal in vivo models, whereas evidence from patients in this regard is scarce, even though the available clinical data appears concordant. When VEGF gene transfer showed no difference in the restenosis rate in a group of PTCA patients, it led to significantly increased myocardial perfusion [84]. Similarly, in a cohort of human lower limb PTA patients treated with VEGF gene transfer in which restenosis per se was not evaluated, it resulted in increased vascularity and better clinical outcomes compared to the control groups [85].

### 3.4. VEGF-Releasing Materials

Besides investigations focusing on the effects of endogenous VEGF and induced VEGF, eluting stents and stent-grafts act as a third pillar in the study of the growth factor and intimal hyperplasia. Early VEGF-eluting stents could increase endothelialization in vitro [86] but not accelerate re-endothelialization or inhibit restenosis in vivo; however, they show reduced thrombosis rates and associated lumen reduction [87]. Newer drug-eluting stents with multiple layer coatings were able to accelerate re-endothelialization and inhibit thrombosis, inflammation and in-stent restenosis, for example through glycoprotein IIIa monoclonal antibody and VEGF121 loading on the inner coating of 316L stainless steel stents with rapamycin loaded into the outer layer [88], or by a polyphenol–polyamine surface that combines the biological functions of nitric oxide and VEGF [89]. Another approach through heparin- and VEGF-loaded nanofiber stent-grafts and nanoplatform stents also showed promising effects by local anticoagulation and induction of rapid endothelialization to counter post-interventional lumen loss [90,91]. Recently, combinations of VEGF and paclitaxel [92] or rapamycin (on a drug-coated balloon rather than a stent) [93] showed promising results regarding the minimization of neointimal hyperplasia.

Instead of growth-factor loading of the stent, a cell-based approach might hold even greater promise. Stents coated with human umbilical vein endothelial cells transfected with VEGF repeatedly promoted reduced neointimal hyperplasia, promoted endothelialization and reduced in-stent restenosis [94,95]. While the coating of stents with VEGF secreting parental umbilical cord blood-derived mesenchymal stem cells leads to more severe restenosis, the combined VEGF and hepatocyte growth factor secretion by these cells shows reduced restenosis rates and enhanced re-endothelialization [96]. Transplantation of endothelial progenitor cells either directly at the injured site or by intravenous application had similarly positive effects mediated at least partially by an increase in endoluminal VEGF [97,98]. These findings further indicate the beneficial effects of endoluminal VEGF regarding re-endothelialization and ISR.

### 3.5. Other Members of the VEGF Family

In cholesterol-fed male New Zealand White rabbits, intimal hyperplasia was induced in the carotid arteries, and subsequently adenoviral vectors encoding a VEGF-E chimera were transferred to the adventitia of the vessel. It could be shown that the periadventitial delivery of the VEGF-E chimera gene significantly increased intimal hyperplasia, medial SMC proliferation, adventitial angiogenesis and macrophage infiltration. The neointimal lesions mainly consisted of SMCs and macrophages. VEGF-E is a specific ligand for VEGFR-2 and its binding in VSMCs induces MCP-1 expression through the PKC/NFκB pathway. MCP-1 enhances SMC proliferation and increases monocyte/macrophage recruitment. The recruited macrophages produced more MCP-1 and VEGFR-2 ligands like VEGF. The VEGFR-2-induced adventitial angiogenesis aggravated neointimal formation [99]. Bhardwaj and colleagues also showed in a similar model that adventitial adenovirus delivery of VEGF-A and VEGF-D increased intimal hyperplasia, which correlated with the presence of MMP-2 and -9 [100]. However, in balloon-injured aortas of New Zealand White rabbits, intravascular adenovirus-mediated VEGF-C gene transfer reduced the intima/media ratio compared to VEGF-A-treated rabbits. Although VEGF-A rabbits also had a reduced intima/media ratio compared to the control group, the beneficial effects are attributed to VEGFR-2 [101].

## 4. Discussion

In 2004, three different studies published in Circulation addressed the role of vascular endothelial growth factor (VEGF) in restenosis after percutaneous transluminal angioplasty and the associated vascular injury, resulting in controversial conclusions [38,65,83]. This prompted a fourth article in the very same issue: a review by Shiojima and Walsh on the discussed topic, which could not answer the diverging outcomes but shed light on the different approaches of the studies and the complex nature of angiogenesis, restenosis and the role and function of VEGF amidst this process [102]. Two decades have passed since the publication of the aforementioned articles, and research regarding VEGF and its effects on intima thickening continues up to today, even though no scientific consensus has been established so far. Here, a systematic review of the literature concerning VEGF and its role in intimal hyperplasia after arterial intervention-mediated injuries was conducted to further address this question.

**Figure 3:**
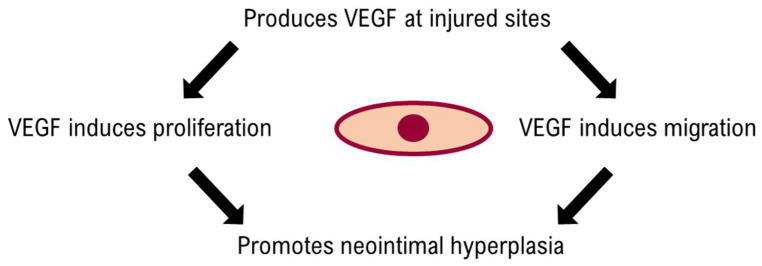
Vascular endothelial growth factor (VEGF) and its relationship to vascular smooth muscle cells.

**Figure 4:**
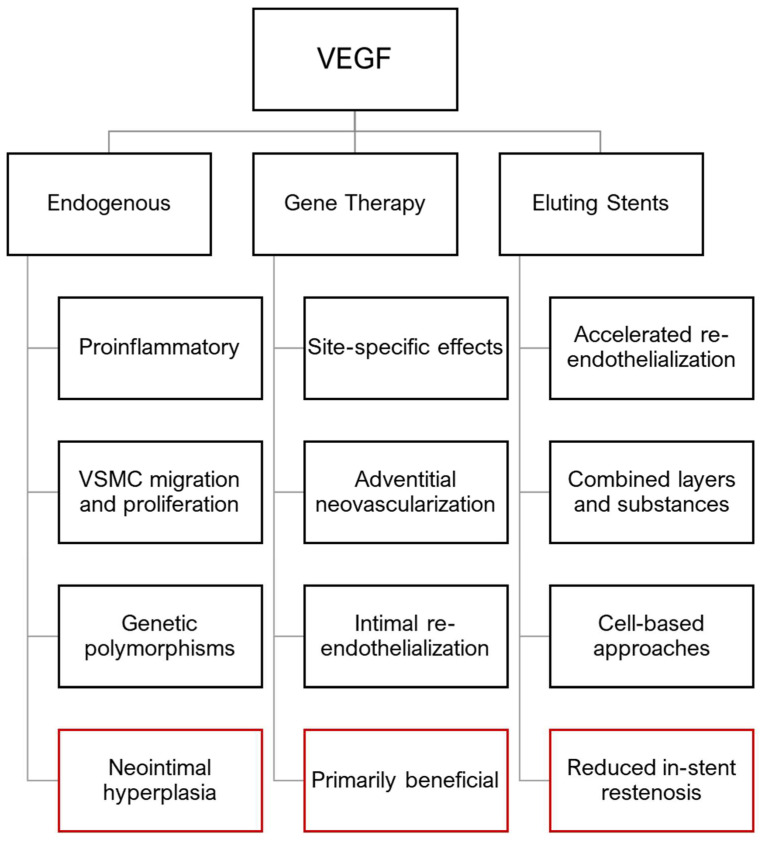
Summary of the primary effects of VEGF in intimal hyperplasia in arterial injuries based on different sources.

Endovascular catheter interventions inevitably lead to endothelial damage that further promotes inflammation, cell proliferation and thrombosis, resulting in luminal occlusion, which sometimes is a desired effect, as in mechanochemical endovenous ablation in chronic venous disease [103]. It is a physiological response to vascular injury. However, in arterial interventions this is commonly considered an adverse effect, especially in coronary and peripheral artery disease. A main contributor to post-interventional stenosis is neointimal hyperplasia. While several factors such as platelet aggregation, leukocyte chemotaxis, collagen deposition and myofibroblast recruitment contribute to intimal hyperplasia, vascular smooth muscle cell proliferation and migration in the absence of an intact endothelium are the main driving force [104,105,106]. Arterial injury leads to autocrine production of VEGF-A, enhanced expression of VEGF-R 1 (Flt-1) and 2 (Flk-2/KDR) and MCP-1 production in VSMCs, which in turn promotes their proliferation and migration and the recruitment of monocytes that secrete additional VEGF (see Figure 3) [107]. Despite a few contrary reports, it is generally accepted that endogenous VEGF is promoting intimal hyperplasia by its effects on VSMCs and proinflammatory attributes. The potential role of genetic VEGF polymorphisms (among other growth factors) on intimal hyperplasia occurrence could help to explain the observed clinical outcome differences in supposedly equally-treated patients [60,61,62] (see Figure 4).

Contradicting data regarding VEGF and neointimal hyperplasia mostly stems from gene therapy applications. It has been previously discussed that this might be based on different experimental procedures or the extent of vascular injury [108]. However, while both factors could be of importance, it additionally seems reasonable to argue that concerning VEGF and neointimal hyperplasia, as in business, location is key. Luminal VEGF application, either through gene transfer or growth factor-releasing stents, increases re-endothelialization and thereby attenuates neointimal hyperplasia. Adventitial or periadventitial VEGF promotes adventitial neo-vascularization and mimics endogenous VEGF and ameliorates its effects, which can ultimately lead to increased neointimal hyperplasia. A summary of the findings is presented in Figure 4. Future research could focus on combined applications of these findings, such as the combination of intraluminal VEGF administration through eluting stents and (peri)adventitial VEGF-R 1 and/or 2 gene transfer, to combat neointimal hyperplasia and restenosis.

Gaps in the evidence, and therefore a potential for further investigative studies regarding the topic, are based on a relatively high share of cell culture and animal models in the current literature. Furthermore, most projects concerned with human patients are based on coronary interventions. Even though there is no indication that human peripheral arteries would lead to utterly contrary results compared to coronary arteries or the aorta, it has been previously discussed that results can vary depending on the applied model, especially when focusing on animal models [102,107,108]. A recent systematic review on VEGF modulation in peripheral arterial disease resulted in a similar conclusion amidst a generally positive association of VEGF and decreased luminal stenosis in PAD studies [109]. Nevertheless, even if, in general, reactions to both endogenous and exogenous VEGF are anticipated to repeat in the peripheral vascular system, smaller deviations, for example in dose-dependency, could be significant for future therapeutic use. Understanding the effects of VEGF-A on intimal hyperplasia is crucial in order to optimize endovascular treatment strategies as well as balloon and stent (graft) designs.

## Data Availability

Data can be made available upon reasonable request to the corresponding author.

## Supplementary Materials

The following supporting information can be downloaded at: https://www.mdpi.com/article/10.3390/jcm14176184/s1, Table S1: The table depicts relevant features of each study included in the main portion of the systematic review.
