# Supplementary material for: The Role of VEGF in Intervention-Mediated Injuries: Neointimal Hyperplasia and In-Stent Restenosis
Source: J Clin Med. 2025 Sep 1;14(17):6184. doi: 10.3390/jcm14176184 (PMC12429427; doi:10.3390/jcm14176184)
Supplement: Supplementary file 1 [file jcm-14-06184-s001.zip › jcm-3765105-supplementary.pdf]

Supplement

Supplementary Table 1. The table depicts relevant features of each study included in the main portion of the systematic review. “Effect” refers to the reported effect of VEGF on neointimal hyperplasia in the respective study and model.

First author	Ref.	Year	Main model	Subsection	Effect
Zhao Q	37	2004	Mice	Endogenous	Promotion
Ohtani K	38	2004	Rabbits, rats, mice	Endogenous	Promotion
Li XD	39	2012	Rats	Endogenous	Promotion
Koga J	40	2009	Mice	Endogenous	Promotion
Shibata M	41	2001	Pigs	Endogenous	Promotion
Tian S	42	2015	Rabbits	Endogenous	Inhibition
Malabanan K	43	2008	Rats	Endogenous	Promotion
Katsaros KM	44	2014	Patients	Endogenous	Promotion
Yin J	45	2017	Patients	Endogenous	Inhibition
Dichtl W	46	2006	Rats	Endogenous	Promotion
Lim SY	47	2007	Pigs	Endogenous	Promotion
Gao JQ	48	2014	Pigs	Endogenous	Promotion
Kochiadikis GE	49	2008	Patients	Endogenous	Promotion
Christoph M	50	2022	Mice	Endogenous	Promotion
Kazmierczak E	51	2014	Patients	Endogenous	Promotion
Li XD	52	2020	Rats	Endogenous	Promotion
Wang J	53	2018	Pigs	Endogenous	Promotion
Mota L	54	2024	Rats	Endogenous	Promotion
Stefanidis C	55	2008	Patients	Endogenous	Promotion

Sun S	56	2018	Rats	Endogenous	Promotion
Krasinski K	57	1997	Rats	Endogenous	Inhibition
Nanobashvili J	58	2004	Rabbits	Endogenous	Promotion
Gao M	59	2022	Rats	Endogenous	Promotion
Osadnik T	60	2017	Patients	Endogenous	Polymorphism
Osadnik T	61	2016	Patients	Endogenous	Polymorphism
Bagyura Z	62	2017	Patients	Endogenous	Polymorphism
Liu Z	63	2024	Rats	Endogenous	Inhibition
Pei CZ	64	2020	Rats	Endogenous	Inhibition
Khurana R	65	2004	Rats	Gene therapy	Promotion
Pels K	66	2004	Pigs	Gene therapy	Inhibition
Deiner C	67	2006	Pigs	Gene therapy	Inhibition
Asahara T	68	1996	Rabbits	Gene therapy	Inhibition
Hytönen JP	69	2018	Rabbits	Gene therapy	Inhibition
Van Belle E	70	1997	Rabbits	Gene therapy	Inhibition
Walter DH	71	2004	Rabbits	Gene therapy	Inhibition
Ye W	72	2019	Rabbits	Gene therapy	Inhibition
Wei F	73	2002	Rabbits	Gene therapy	Inhibition
Liu Q	74	2004	Rabbits	Gene therapy	Inhibition
Gao F	75	2006	Pigs	Gene therapy	Inhibition
Zhang T	76	2016	Rabbits	Gene therapy	Inhibition
Hao X	77	2022	Mice	Gene therapy	Inhibition
Dulak J	78	2005	Rabbits	Gene therapy	Promotion
Buchwald AB	79	2006	Pigs	Gene therapy	Inhibition
Paul A	80	2012	Dogs	Gene therapy	Inhibition
Yang J	81	2013	Pigs	Gene therapy	Inhibition

Xie H	82	2015	Rabbits	Gene therapy	Inhibition
Hutter R	83	2004	Mice	Gene therapy	Inhibition
Hedman M	84	2003	Patients	Gene therapy	Neutral
Mäkinen K	85	2002	Patients	Gene therapy	Neutral
Swanson N	86	2003	In vitro	VEGF eluting	Neutral
Swanson N	87	2003	Rabbits	VEGF eluting	Neutral
Hu T	88	2017	Rabbits	VEGF eluting	Inhibition
Zhang B	89	2022	Rabbits	VEGF eluting	Inhibition
Wang J	90	2015	In vitro	VEGF eluting	Inhibition
Tan J	91	2020	In vitro	VEGF eluting	Inhibition
Karaagac E	92	2021	Rabbits	VEGF eluting	Inhibition
Lai YX	93	2023	Rabbits	VEGF eluting	Inhibition
Tang C	94	2011	In vitro, rabbits	VEGF eluting	Inhibition
Wu X	95	2016	Rabbits	VEGF eluting	Inhibition
Chang HK	96	2018	Pigs	VEGF eluting	Inhibition
Wang W	97	2021	Rats	VEGF eluting	Inhibition
Sun H	98	2023	Rats	VEGF eluting	Inhibition
